# Identification of a Known Mutation in Notch 3 in Familiar CADASIL in China

**DOI:** 10.1371/journal.pone.0036590

**Published:** 2012-05-18

**Authors:** Zhen-Xuan Tan, Fei-Feng Li, You-Yang Qu, Ji Liu, Gui-Rong Liu, Jin Zhou, Yu-Lan Zhu, Shu-Lin Liu

**Affiliations:** 1 Genomics Research Center, Harbin Medical University, Harbin, China; 2 Neurology Department of The Second Affiliated Hospital, Harbin Medical University, Harbin, China; 3 Genetic Detection Center of The First Clinical College, Harbin Medical University, Harbin, China; 4 Department of Microbiology and Infectious Diseases, University of Calgary, Calgary, Canada; University of Florida, United States of America

## Abstract

**Background:**

Cerebral autosomal dominant arteriopathy with subcortical infarcts and leukoencephalopathy (CADASIL) is an inherited disease leading to recurrent ischemic stroke and vascular dementia. Numerous mutations in the 23 exons of the NOTCH3 gene have been reported to cause CADASIL in Caucasian populations, but the full spectrum of genetic changes leading to this disease is yet to be known and, especially, very few reports are available on CADASIL in Asian populations.

**Methods and Results:**

We genotyped members of a 5-generational Han Chinese family with CADASIL patients and identified an R133C mutation in the NOTCH3 gene. Clinical analysis demonstrated that the penetrance of the mutation was not complete. Five of the mutation carriers, not exposed to the known vascular risk factors, did not show any clinical feature of CADASIL, suggesting the importance of environmental factors to the development of this disease.

**Conclusions:**

Members of a 5-generational Han Chinese family with CADASIL patients had an R133C mutation in the NOTCH3 gene but only individuals exposed to known vascular risk factors developed CADASIL.

## Introduction

Cerebral autosomal dominant arteriopathy with subcortical infarcts and leukoencephalopathy (CADASIL) is an inherited disease with mutations in the NOTCH3 gene [1], [2], [3]. This disorder has been found in many race-ethnicities, with most reported cases coming from European Caucasian families [2], [4], [5]. To date, very few cases in Asian families have been reported [6], which however may not necessarily indicate that the disease is rare in Asia.

The main clinical feature of the disease is the disfunctioning of the central nervous system (CNS), characterized by recurrent ischemic attacks or strokes, migraine, cognitive impairment, dementia and psychiatric disturbances [7]. The mean onset age is around 45 years old, ranging from 30 to 70 [2], [4], [5]. About 85% of symptomatic CADASIL patients have ischemic attacks or stroke and 22–64% show migraine, which may begin early during childhood or adolescence but mostly during the third decade [2], [4], [6]. Many CADASIL patients also show cognitive decline, dementia and psychiatric symptoms [6]. In addition to these common CNS symptoms and signs, some less frequent manifestations of the disease have also been reported, such as epilepsy, transient disturbances of consciousness, visual impairment, and hemorrhagic strokes [7], [8], [9], [10].

A large number of mutations in the 23 exons of the NOTCH3 gene have been reported to be associated with CADASIL [1], [5], [7], [11], [12], [13], [14]. However, the full spectrum of genetic changes leading to this disease is yet to be known and, especially, very few reports are available on CADASIL in Asian populations. Here, we report an R133C mutation on exon 4 of the *NOTCH3* gene in members of a 5-generational Han Chinese family and describe the unusual clinical manifestations of the disease in this family.

## Results

### Clinical Data

The proband was a 60-year-old male (ΙΙ:5; Figure 1), whose symptoms began at the age of 47. The main clinical manifestations included mild dysarthria and left central facial and tongue paralysis. Lower jaw reflex was brisk, bilateral palm-chin reflex was brisk, bilateral gag reflex was slow, limb tendon reflexes were brisk, with the lower limbs being pronounced. Left rotation movement was clumsy, and the Romberg sign was brisk. The patient had a right hemiparesis.

**Figure 1:**
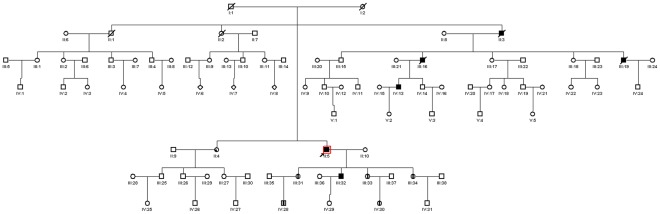
Pedigree of a 5-generational Chinese Han family affected with CADASIL. Squares and circles indicate males and females, respectively. Filled symbols denote affected status. Normal individuals are shown as empty symbols. Mutation carriers without evident clinical features are indicated by a circle with a vertical line in the middle (III:31, III:33, III:34, IV:30) or a square with a vertical line in the middle (IV:28). II:4 had a large area cerebral infarction but was not a CADASIL patient.

**Figure 2:**
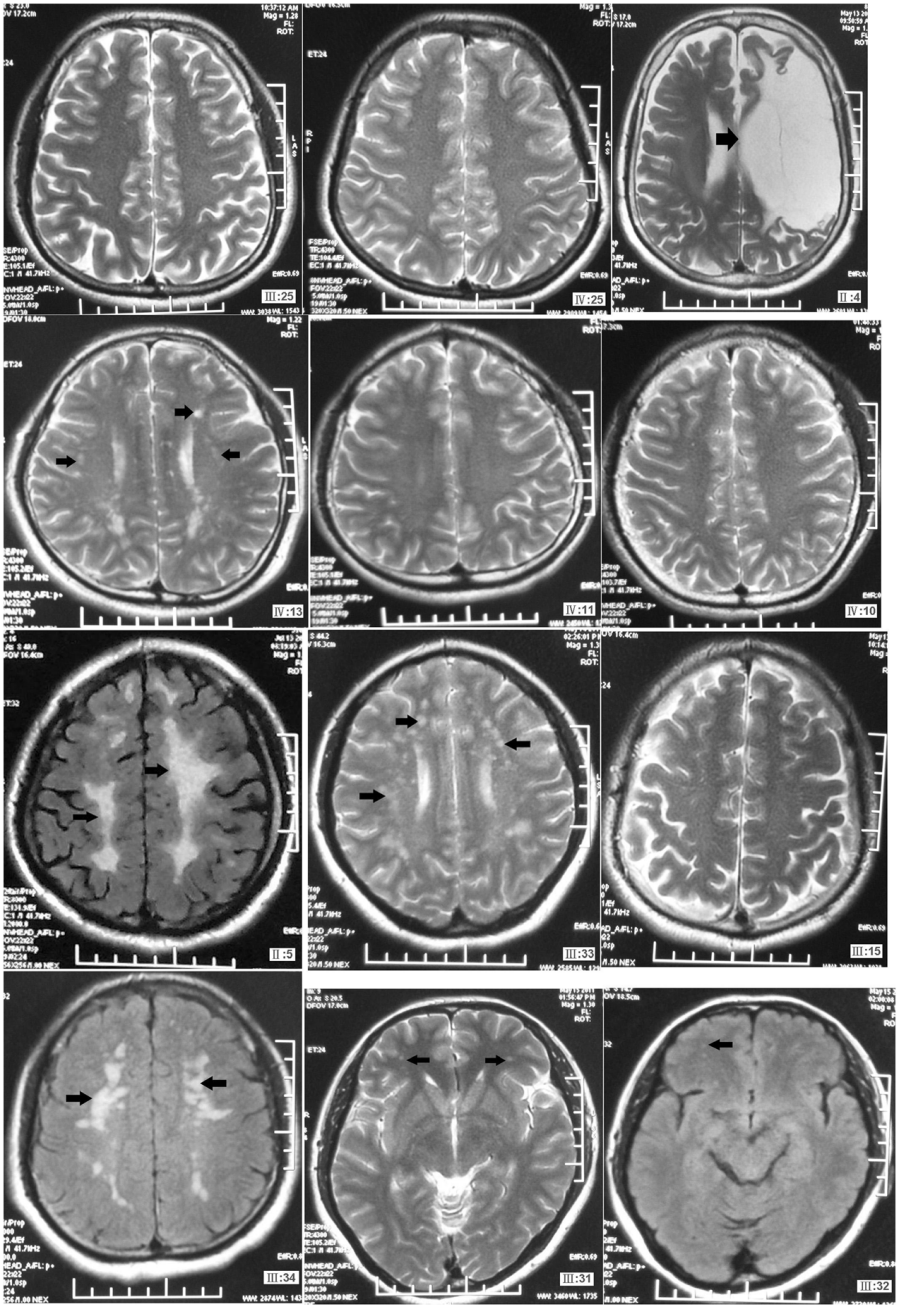
Cerebral MRI examination in the CADASIL family members. ΙΙ:5, the proband, was a 60-year-old male affected family member. ΙΙΙ:31, ΙΙΙ:32, ΙΙΙ:33 and ΙΙΙ:34 were the children of the proband (ΙΙ:5). ΙΙ:5 and ΙΙΙ:32 showed evident clinical features, but ΙΙΙ:31, ΙΙΙ:33 and ΙΙΙ:34 did not show any clinical feature, although they were all mutation carriers (see the long T1 and T2 signals). ΙΙΙ:25, ΙΙΙ:15, ΙV:25, ΙV:10 and ΙV:11 were healthy core family members. ΙΙΙ:4 is a patient with l massive cerebral infarction, who did not have the R133C mutation.

The MRI examination results showed long T1 and long T2 signals on the white matter around the ventricles, and punctate long T1 and long T2 signals in the brainstem (Figure 2). His intelligence score was normal. Urine routine test, blood glucose, blood lipids, liver and kidney functions, blood homocysteine levels, ECG and abdominal ultrasound results were all normal. This 5-generational family included 6 affected individuals, 72 unaffected individuals, 5 mutation carriers who did not show symptoms and one large area cerebral infarction patient (ΙΙ:4), who was not a CADASIL patient and did not have the mutation. The main clinical features of the proband and the other affected individuals in the family were summarized in Table 1.

**Table 1:** Clinical features of the affected individuals.

Patient	Sex	Age	Onset Age	Migraine	Memory	MRI Examination	Vascular Risk Factors	Other Symptom
ΙΙ:4	F	67	48	–	Severe	Gliosis in the lefttemporal lobe, foreheadand parietallobe; massivecerebralinfarction	–	Ineffective activity ofright limbs accompaniedby verbal clumsinessfor over 20 years;obvious memorydecline; nodysphagia or dysuria.
ΙΙ:5	M	60	47	–	Mild	Long T1 andT2 signals shownon the whitematter around theventricles; punctuatelong T1 andlong T2signals in the brainstem;CADASIL patient.	Smoking	Unsteady gait for2 months, gettingworse for1 week; numbnessin hands andfeet; languageunclear; ataxia;lower extremityweakness.
ΙΙΙ:32	M	40	30	+	Mild	Punctate longT1 and longT2 signals onthe temporal lobes;bilateral basal gangliaand corona radiateand high-intensitysignals of FLAIRsequence; CADASIL patient.	Smoking	Numbness in handsand feet; nosymptoms ofineffective activityof the limbs,memory declineor headache.
ΙV:13	M	31	20	+	Mild	Punctate long T1and T2 signals onright hemisphere ofcerebellum, bilateral temporallobes, bilateral basalganglia and coronaradiate, high-intensitysignals of FLAIRsequence; CADASILpatient.	Hypertension	Drowsinessaccompanied byparoxysmal hemicrania forover 10 years;headache during thecourse of thedisease; MMSEscore 29.
ΙΙΙ:31	F	41	–	–	–	Abnormal punctate longT1 and T2signals on bilateralfrontal lobes, slightlyhigher signal intensityof lesions onFLAIR;CADASIL patient.	–	–
ΙΙΙ:34	F	36	–	–	–	Abnormal punctate long T1 and long T2 signal shown on bilateral frontal lobe; CADASIL patient.	–	–
ΙΙΙ:33	F	36	–	–	–	Punctate long T1and long T2signals on bilateraltemporal lobes,bilateral basal gangliaand corona radiate;CADASIL patient.	–	–

### NOTCH3 Mutation

**Figure 3:**
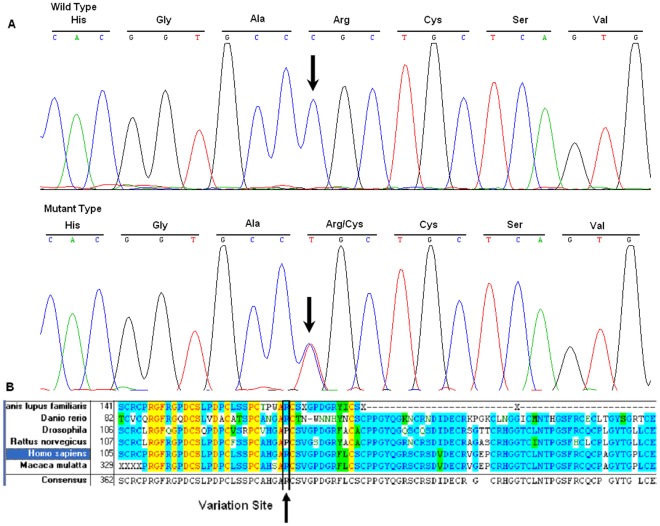
Analysis of the DNA and protein sequences. DNA sequence chromatogram of the R133C mutation in *NOTCH3* and multiple-sequence alignment of NOTCH3 protein family. The C→T transition at position 475 resulting in the R133C mutation is located within a highly conserved region. A: DNA sequence chromatogram of the unaffected family members and ΙΙΙ:4; B: DNA sequence chromatogram of the affected family members and ΙΙΙ:31 and ΙΙΙ:33; C: multiple-sequence alignment of NOTCH3 protein family.

Sanger sequencing of the amplified fragments of *NOTCH3* in all affected individuals identified a single base alteration, 475C→T (Figure 3A, B), in exon 4 of the *NOTCH3* gene (GI:4854) located at 19p13.2-p13.1, resulting in the substitution of Arg to Cys at codon 133 (R133C). The remainder of the coding sequence showed no other changes. Further sequence analysis revealed that all affected members in this family carried the 475C→T mutation, although 5 mutation carriers did not show symptoms. The other individuals in this family did not carry this mutation, and the mutation was not present in 100 normal controls.

### Conservation of the Protein in Evolution

Comparison of NOTCH3 protein sequences from six mammalian species by multiple-sequence alignment analysis showed that the 133Arg residue was located in a highly conserved region of the protein (Figure 3C).

## Discussion

In this study, we identified a mutation, 475C→T (R133C), in the NOTCH3 gene in a 5-generational Han Chinese family with CADASIL patients. This mutation co-segregated with the disease phenotype in all affected individuals except ΙΙΙ:4, who was a patient with massive cerebral infarction but not a CADASIL patient. The mutation was not present in 100 normal control subjects. Five members of the family, ?:31, ?:33, ?:34,108 ?28 and ?30, were mutation carriers but did not show any clinical feature of CADASIL. The result of multiple-sequence alignments showed that Arg133, mutation of which was previously described by Joutel [15] and Mykkanen [16], was a conserved residue, indicating its importance for normal NOTCH3 function.

In this family, the penetrance rate was not 100% in the mutation carriers, but MRI examination showed that all the mutation carriers had long T1 and T2 signals within the temporal lobes and high-intensity signals of FLAIR sequence. However, ΙΙΙ:31, ΙΙΙ:33 and ΙΙΙ:34 did not show any clinical feature of CADASIL. One fact worth noting is that these three family members had not been exposed to any known vascular risk factors, such as smoking, drinking, and hypertension. On the other hand, ΙΙΙ:5, ΙΙΙ:32 and ΙV:13, who showed very evident clinical features, had obviously contacted vascular risk factors, such as smoking or hypertension. The genotype–phenotype correlation in CADASIL has not been clarified. Although some data support genotype–phenotype correlation in CADASIL [7], [13], [17], [18], the mutation found in patients of the family did not have specific effects on the expressivity of the disease. Some authors report that vascular risk factors [19], [20] or other unexplored factors may influence the phenotypic variability and lead to atypical features of the CADASIL patients, which may explain why ΙΙΙ:31, ΙΙΙ:33 and ΙΙΙ:34 did not show any clinical feature of CADASIL. However, correlations between vascular risk factors and expressivity of the disease would require larger scales of study involving many more patients and controls for a conclusion.

NOTCH3 (N3) is one member of the Notch receptor superfamily, which regulates cell fate during embryonic development [21] and is predominantly expressed in vascular smooth muscle cells (VSMC) in adulthood [1], [22]. Appropriately half of identified CADASIL-related mutations are located on exons 2–4 [7], [11], which encode the extracellular domain of N3 (N3^ECD^) within epidermal growth factor-like (EGF-like) repeat domains [15], [23], [24]. Modular structures show that six highly conserved cysteine residues within these domains stabilize the domain [25]. Dichgans and colleagues, by blocking or facilitating disulfide bridge formation, found that multimerization of N3, at least in part, depends on disulfide bridges and unpaired cysteine residue might make CADASIL-mutated N3^ECD^ more susceptible to multimerization in higher order complexes [26].

In summary, this study identified the 475C→T (R133C) mutation in the *NOTCH3* gene in a 5-generational Han Chinese family with CADASIL. Five family members were not exposed to any vascular risk factors and they did not show any clinical feature of CADASIL. Vascular risk factors may play a vital role in the development of CADASIL.

## Materials and Methods

### Affected and Unaffected Individuals in the Family

We ascertained a 5-generational Chinese Han family with non-syndromic CADASIL (see Figure 1) at the Second Affiliated Hospital of Harbin Medical University, Harbin, China. Informed consent was obtained from each participant, consistent with the Declaration of Helsinki. We recorded their medical history in detail. Physical and MRI examination was carried on each of the family members. Genomic DNA was extracted from peripheral blood leukocytes using standard protocols.

### DNA sequencing

Individual exons of *NOTCH3* were amplified by PCR using primer pairs shown in Table S1. The PCR products were sequenced on an ABI3130 Automated Sequencer.

### Multiple Sequence Alignment

From the NCBI website (http://www.ncbi.nlm.nih.gov/), the NOTCH3 protein sequence of various species were obtained and, by using the Vector NTI software, multiple-sequence alignments of NOTCH3 proteins were carried out.

## Supporting Information

Table S1 **PCR primers and PCR product sizes for NOTCH3 sequence analysis.**
(DOC)
