# Supplementary material for: Identification of a Known Mutation in Notch 3 in Familiar CADASIL in China
Source: PLoS One. 2012 May 18;7(5):e36590. doi: 10.1371/journal.pone.0036590 (PMC3356370; doi:10.1371/journal.pone.0036590)
Supplement: Table S1 — PCR primers and PCR product sizes for NOTCH3 sequence analysis. (DOC) [file pone.0036590.s001.doc]

S-Table 1: PCR primers and PCR product sizes for NOTCH3 sequence analysis

| **Primer pair** | **Exon** | **Forward primer** | **Reverse primer** | **Size**  **(bp)** | **Tm**  **(**℃**)** |
| --- | --- | --- | --- | --- | --- |
| 1 | Exon1 | GTTCTGCCCGCGTCTCAC | TCCCCGACCCTGGTTCCT | 501 | 64.0 |
| 2 | Exon2 | GTGGGAACTGTGGATGGG | AGAGGCAGAGGGAGAAGA | 317 | 57.3 |
| 3 | Exon3 | TCTTGTGTGTATCTTTGT | AGGGTGAGTTTAGGACTG | 275 | 52.6 |
| 4 | Exon4 | GGGGAGGTAGTCGGGGGT | AGGGAAGGGGGCAAGGAT | 483 | 59.9 |
| 5 | Exon5 | CCAGAGGGAGCGGGGAGG | ATGATTACAGGCATGAGC | 431 | 53.7 |
| 6 | Exon6 | GGGCAACAAGAGCGAAAC | CACTGGCACAGGAAGGAG | 451 | 55.6 |
| 7 | Exon7 | GTGGACGAGTGCTCTATC | CTTACACCCCATTCTGCT | 395 | 55.6 |
| 8 | Exon8 | AAGGCTCGGGGGATTTGT | CCCACCCTGGAGTTTTTG | 289 | 56.1 |
| 9 | Exon9 | GGGAACCTGTAAAACCAC | TCCACCCCCCAACTCTGT | 427 | 53.9 |
| 10 | Exon10 | AAGTGGGGGGTGGGGGGT | TCCAGGTGTGCTGTTTCT | 427 | 56.5 |
| 11 | Exon11 | AACAGCACACCTGGAGGG | GCAAAGATACGGGCAAAA | 291 | 55.6 |
| 12 | Exon12 | GCGTTATGAAAAAGAGGT | CTGGCAGGGGAAGGTAGT | 391 | 54.5 |
| 13 | Exon13 | CCTCAGACACTGCCCCCT | GATTGAAAGCAAAAAAGA | 323 | 54.3 |
| 14 | Exon14 | CTCTACTCTCTCCTCCCG | CAGCTCTGTCCTTCCAAG | 429 | 54.7 |
| 15 | Exon15 | GATAAAGGAGGGGACGAG | AGTGGGTACCAAGCTGAG | 517 | 56.3 |
| 16 | Exon16 | GCCGAGATAAGGGTCAGG | TGTGGGGTGGGGGGTAGT | 423 | 58.5 |
| 17 | Exon17 | GTTTCTCCCAGACTACCC | CAGCCACACACCCCATCA | 329 | 55.4 |
| 18 | Exon18 | GTCAGTGATGAGACCTTG | GAGACATACCCATACCAA | 447 | 53.0 |
| 19 | Exon19 | ACATCTGTGTGTGTCCTT | CCGCAGTCATCCTCATTA | 357 | 52.3 |
| 20 | Exon20 | CCCGCTATCTCTGCTCCT | GCCCCTTCGCCAACGCTT | 359 | 57.3 |
| 21 | Exon21 | GTTTCCGTTGCCTTTGTC | CCCTTCGATGTCTCCCCT | 343 | 56.8 |
| 22 | Exon22 | CCTCTCTCCCCTTGACTC | CCCACGGACAAACAGACT | 737 | 59.3 |
| 23 | Exon23 | CTGTACTCTACGGTGTGA | GAGGCATTTTTTGCTTGT | 569 | 54.4 |
| 24 | Exon24 | GTGCAGTGGGGTTGGATG | ACGCTGGGTTCTGGAGGC | 467 | 59.0 |
| 25 | Exon25 | CGGTGGAGCGCCTGGACT | GCTGGGAAGGAGGATTTG | 495 | 59.0 |
| 26 | Exon26 | CCAAATCCTCCTTCCCAG | TCACGCCCATCATCCACT | 241 | 56.4 |
| 27 | Exon27 | ACAAATAAAACACCAAAC | CAGAAATAACCTCTCACA | 385 | 51.4 |
| 28 | Exon28 | ATTTTTCCATGTGTCCCA | GTATTCCCATATATCCCC | 535 | 53.1 |
| 29 | Exon29 | CTTCATGGGACTTAGGGG | GTGAGCTTCAGTGATTGG | 335 | 52.9 |
| 30 | Exon30 | AGGTGATGGATGAATGTG | TTGAGGCCAGGGAGGAAG | 435 | 54.7 |
| 31 | Exon31 | AGGACATCGTGCGCTTGC | ACAGCCACAGGGTTCAGC | 451 | 60.0 |
| 32 | Exon32 | AGGCCGGGCGGGTCTAGG | AGGACGGGGGTCTCTTTA | 587 | 59.3 |
